# Course of Pregnancy in a Woman With Familial Chylomicronemia Syndrome Treated With Plozasiran, a Small Interfering RNA Against ApoC3

**DOI:** 10.1002/jmd2.70052

**Published:** 2025-12-04

**Authors:** Miriam Larouche, Diane Brisson, Nathalie Roy, Claudy Grenon, Paul Poirier, Ma'an Muhsin, Daniel Gaudet

**Affiliations:** ^1^ Department of Medicine Université de Montréal and ECOGENE‐21 Chicoutimi Canada; ^2^ Faculty of Pharmacy, Université Laval Quebec City Canada; ^3^ Institut Universitaire de Cardiologie et de Pneumologie de Québec, Université Laval Quebec City Canada; ^4^ Arrowhead Pharmaceuticals, Inc. San Diego California USA

**Keywords:** APOC3, familial chylomicronemia syndrome, persistent chylomicronemia, plozasiran, pregnancy, siRNA

## Abstract

Persistent chylomicronemia is associated with severe hypertriglyceridemia (sHTG) and plasma triglycerides (TG) levels sustainably > 10 mmol/L (880 mg/dL) despite lipid lowering therapies. The main risk of persistent chylomicronemia is acute pancreatitis (AP). During the second and third trimester of pregnancy, TG levels significantly increase, which represents a serious risk of AP in women with preexisting chylomicronemia. New emerging therapies such as plozasiran, a GalNAc‐conjugated small interfering RNA (siRNA) against ApoC3, are developed to manage persistent chylomicronemia, but no data are currently available on their safety and efficacy during pregnancy. We report herein the case of a woman with persistent chylomicronemia randomized in the PALISADE study to receive plozasiran 25 mg quarterly, who had an unplanned pregnancy during the clinical trial. The 34‐year‐old patient received one dose of plozasiran 8 weeks before conception and the experimental treatment was ceased afterwards. The pregnancy went well, TG levels did not rise above 10 mmol/L (880 mg/dL) during the whole pregnancy, even during the last two trimesters where TG levels usually increase two‐ to four‐fold from baseline and she did not experience any AP episode. She delivered a healthy baby at 39 weeks. This case suggests that plozasiran might be safe for the mother and the fetus and could prevent incremental pregnancy‐driven TG elevation and occurrence of AP in women with sHTG. This is consistent with the long duration of action and hepatic half‐life of plozasiran in clinical studies where TG levels remained sustainably lower than baseline > 9 months after the last injection.

## Key Points

- Persistent chylomicronemia (triglyceride [TG] values > 10 mmol/L or 880 mg/dL) represents a high risk of acute pancreatitis (AP), especially during pregnancy.
- Plozasiran, a GalNAc‐conjugated ApoC3 siRNA that is injected subcutaneously every 3 months in patients with persistent chylomicronemia, has a short half‐life in the bloodstream (3–4 h) but a long half‐life in the liver cells.
- We report the case of a woman with persistent chylomicronemia injected with plozasiran 8 weeks before conception who maintained TG levels significantly below the pancreatitis risk threshold throughout pregnancy.
- The pregnancy went well, and the mother delivered a healthy baby, suggesting that plozasiran has a long duration of action and that its administration a few weeks before pregnancy in women with persistent chylomicronemia might prevent AP while being safe for the fetus. More data are needed to replicate this observation.

## 1 Introduction

The ability to balance maternal and fetal risks and benefits of a pharmacological treatment for a severe health condition during pregnancy represents a clinical challenge [1, 2, 3]. This is especially the case for women with severe hypertriglyceridemia (sHTG) who are at high risk of HTG‐induced acute pancreatitis (AP), as pregnancy further increases triglyceride (TG) levels. Newly developed therapies having a long duration of action, but short bloodstream half‐life, are being developed and have been proven to be effective in lowering TG levels including in patients with severe HTG.

Chylomicronemia is the most severe form of HTG and is characterized by TG levels > 10 mmol/L (880 mg/dL) as a result of fasting accumulation of chylomicrons in plasma [4]. Chylomicronemia can be persistent or transient [5, 6, 7] and its prevalence in the occidental world is estimated at 1:500 [8]. Persistent chylomicronemia is rarer, with an estimated prevalence of 1:5500 [8]. The most severe form of persistent chylomicronemia is called the familial chylomicronemia syndrome (FCS), an ultrarare disorder affecting 1 to 10 individuals per million [9]. FCS can be caused by bi‐allelic combinations of pathogenic variants in the *LPL* gene or genes involved in LPL function such as apolipoprotein C2 (*ApoC2*), *ApoA5*, lipase maturation factor 1 (*LMF1*) or glycosylphosphatidylinositol anchored high‐density lipoprotein binding protein 1 (*GPIHBP1*), or other factors (clinical FCS). However, most often, chylomicronemia results from a combination of genetic susceptibility, comorbidities such as obesity, type 2 diabetes, and/or environmental triggers (life habits, nutritional habits, drugs affecting TG levels, epigenetic modifications) [10].

Chylomicronemia confers a high risk of AP, which importantly increases during pregnancy due to hormonal changes modifying lipid metabolism. These changes exacerbate HTG, which can become life‐threatening with substantial maternal and fetal complications in women with preexisting chylomicronemia. AP prevalence in pregnant women with chylomicronemia is up to 120‐fold higher than what is reported in pregnant women from the general population (0.35%) [11, 12, 13, 14]. AP can be associated with temporary or permanent organ failure, pancreatic necrosis, as well as a significant risk of maternal (21%) and fetal (20%) mortality [15, 16, 17].

In the first two trimesters of pregnancy, fat is directed to storage deposits to be used in later stages of gestation [17]. This is a physiological adaptation to support fetal growth and lactation. Changes in TG concentration are mainly due to the increased levels of estrogens, and to a lesser extent progesterone and human placental lactogen, all together substantially decreasing LPL activity. LPL is the key enzyme hydrolyzing TG from chylomicrons and very‐low‐density lipoproteins (VLDL) [15]. Estrogens also reduce hepatic lipase activity and are associated with an increased TG synthesis and VLDL production by the liver, leading to increased circulating TG levels [18, 19]. Progesterone's impact is less profound than estrogen, but still important as it reduces lipolysis and promotes fat storage, which could lead to increased plasma TG levels. It is also contributing to insulin resistance in pregnancy, together with human placental lactogen. Furthermore, insulin resistance in late pregnancy decreases LPL activity, impairing TG clearance.

In the general population, these modifications in lipid metabolism are well tolerated and have no significant clinical relevance. However, complications can arise in women with sHTG which can lead to AP, hyperviscosity, and potential preeclampsia [17]. The causes of HTG‐induced AP are diverse and not perfectly understood. AP might be caused by increased viscosity of plasma due to chylomicron accumulation, thus inducing ischemia, acidosis and trypsinogen activation [20, 21]. Another hypothesis postulates that TG metabolism induces a higher production of free fatty acids damaging acinar cells in the pancreas [20, 21]. Also, lysophospholipids and phospholipid remodeling modulate chylomicron assembly and could contribute to systemic inflammation [22].

The treatment of chylomicronemia during pregnancy was, until recently, mainly nutritional. It is recommended to adapt the caloric intake to the mother and fetus' needs, and to use medium‐chain TG (MCT) not requiring chylomicron formation to be assimilated [23]. TG‐lowering treatments such as fibrates and omega‐3 can be added to the diet, but data are insufficient to demonstrate their true benefit, and patients with FCS usually do not respond to these agents, due to their effects confined to the LPL‐dependent pathway [17]. Insulin and unfractionated heparin infusions have also been used during pregnancy, but no clear data from controlled clinical studies have been provided yet [24]. Additionally, Watts et al. reported a case of a pregnant woman having developed severe HTG due to the long‐term use of heparin in prophylaxis [25]. Plasmapheresis can be required in some women with a residual HTG after treatment with safe TG‐lowering therapies during pregnancy [26]. Successful pregnancy of a woman using prophylactic plasmapheresis has been described by Pecin et al. [27]. However, this specialized technique is invasive, time‐consuming, and not widely available [27, 28]. Recently, the clinical utility of plasmapheresis in the treatment of HTG‐induced AP has been questioned [29]. Several emerging treatments having long duration of action are developed to manage chylomicronemia, including ApoC3 inhibitors, but data currently available on their safety and efficacy during pregnancy are lacking.

We report herein on a woman with persistent chylomicronemia (FCS) participating in a phase 3 clinical trial (NCT05089084) [30], treated with plozasiran, an ApoC3 small interfering RNA (siRNA), who became pregnant under treatment.

## 2 Case Report

The patient is a 34‐year‐old woman with FCS who met both the genetic and the clinical diagnostic criteria in the PALISADE study [30]. She carries the P234L pathogenic and N318S loss‐of‐function variants in the *LPL* gene and had historical TG plasma concentrations persistently above 10 mmol/L (880 mg/dL) since adolescence. She presented eruptive xanthomas in the past and recurrent episodes of abdominal pain and fatigue. She experienced 2 AP episodes in the last 5 years. She weighed 73.3 kg with a height of 1.55 m (body mass index of 30.5 kg/m^2^). Her first pregnancy occurred in 2020 at age 32 and she delivered a healthy baby at 39 weeks of gestation by cesarean section. She had close monitoring during this pregnancy since she was known to have persistent chylomicronemia. She gained 13.6 kg during the whole pregnancy, developed gestational diabetes and her TG levels rose up to 45 mmol/L (3986 mg/dL) during the third trimester even though she was on a strict diet and received insulin injections to upregulate bioavailable LPL. The pregnancy went well, and she delivered at term a healthy baby weighing 2.7 kg (5.95 lbs) who did not present with signs of failure to thrive. Six months after delivery, the mother had an AP episode with pancreatic necrosis. During that episode, TG levels rose up to 73 mmol/L (6636 mg/dL), approximately 43‐fold above the upper limit of normal [4].

In July 2022, the patient was enrolled in the phase 3 PALISADE study (NCT05089084) with plozasiran, designed with a double‐blind treatment period of 12 months and an open‐label extension of 24 months. She was screened on July 28, 2022, randomized and received her first dose of 25 mg subcutaneously on September 14, 2022. Her TG levels decreased from 24.4 mmol/L (2158 mg/dL) at screening to 0.81 mmol/L (72 mg/dL) 30 days after treatment. The patient has been using Evra (Q3W) since October 2021 and confirmed that there was no interruption in contraception, but that condoms were not consistently used. Eight weeks after randomization, the patient suspected that she was pregnant, and beta‐HCG was measured at 49227 U/L. No further dose of the investigational product was injected during this unplanned pregnancy, but the patient accepted pursuing the clinical trial follow‐up visits.

**FIGURE 1:**
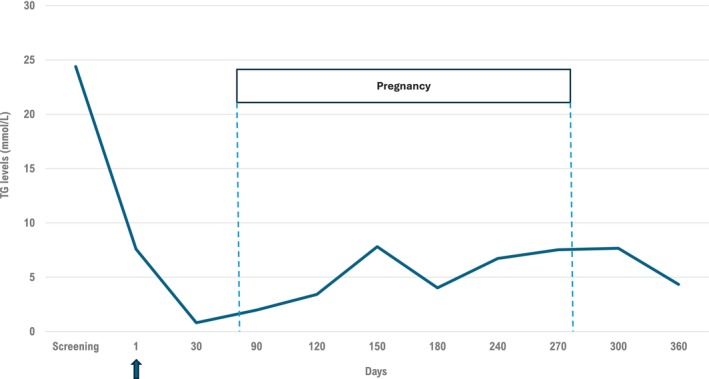
TG levels from screening to the end of the clinical study. Plozasiran was injected (arrow) at randomization (day 1). The unplanned pregnancy was confirmed 8 weeks after injection. Following plozasiran administration, TG levels rapidly decreased and remained below the threshold of AP risk until the end of the clinical study, covering the whole pregnancy and the first postpartum weeks, and resulting in a completed pregnancy, a full‐term healthy baby, with normal weight and developmental milestones.

This second pregnancy progressed well with minor back pain and gestational diabetes reported around the third trimester. The patient delivered a full‐term, healthy female baby by cesarean section after 39 weeks of gestation with a weight of 4.1 kg (9 lbs. 2 oz), length of 47.5 cm. The APGAR (appearance, pulse, grimace, activity, and respiration) score was 3 at 1 min, 8 at 5 min, and 7 at 10 min. The newborn was not premature and did not present signs of fetal distress. The mother had close monitoring of the pregnancy and gained 18.2 kg (40 lbs). She was treated with a strict restrictive fat diet (< 10%–15% of daily calorie intake) as recommended in the latest NLA guidelines [7] and with insulin injections and no AP episode occurred during pregnancy nor in the postpartum period. TG levels were assessed throughout pregnancy through the clinical trial visits. Even though the patient received only one dose of plozasiran 8 weeks preconception, her TG levels never exceeded 10 mmol/L (880 mg/dL) during the entire pregnancy (Figure 1) whereas TG levels increased up to 45 mmol/L (3986 mg/dL) at the end of the first pregnancy. Additionally, the subject experienced no lactation problems and 1 year after giving birth, the baby is reported to be in good health with normal developmental milestones. The baby's 1‐year weight was reported as 9.977 kg (22 lbs) and length of 74 cm which are within the normal age ranges.

## 3 Discussion

This report presents a patient with a successful pregnancy following plozasiran treatment. In this study, the patient received a dose of plozasiran 8 weeks before the beginning of her pregnancy and her TG levels never rose above 8 mmol/L (710 mg/dL) during the entire gestational period, even during the third trimester where TG levels normally increase two‐ to four fold from baseline [17]. TG levels remained below 10 mmol/L (880 mg/dL), the risk threshold for AP [31], suggesting that plozasiran might prevent TG elevation and, consequently, might decrease the occurrence of sHTG‐related AP during pregnancy even when injected weeks before conception.

The phase 3 PALISADE study with plozasiran demonstrated a significant decrease in TG levels (−80%) in patients with persistent chylomicronemia, with a maximal decrease achieved at day 30 [30]. The pregnant woman here described showed a 97% reduction in TG levels at day 30 and a 92% reduction at day 90. In prior studies conducted with plozasiran, it has been estimated that its half‐life in the bloodstream was very short, in the range of 3–5 h [32], with a substantial and sustained efficacy in plasma TG lowering still observable after 24 or even 48 weeks [32, 33, 34]. However, the half‐life of plozasiran in the hepatocytes, the targeted tissue, is longer (several months) due to the use of the RISC system for recycling [35]. This sustained liver efficacy might explain the low levels of TG during the patient's pregnancy even though plozasiran was stopped after the first injection.

The rapid internalization of the hepatocyte‐targeting *N*‐acetylgalactosamine‐conjugated (GalNAc) siRNA in the liver might decrease the odds of off‐target delivery, avoiding long‐term systemic side effects for the mother and decreasing the risk of fetal toxicity through transplacental transfer. In this patient report, no deleterious impacts of plozasiran injected 8 weeks before pregnancy were observed on the development of the fetus, including prematurity or failure to thrive.

Clinical data on the safety and risk/benefit ratio of siRNA therapies in pregnant women is lacking. Two reports of pregnant women previously treated with siRNAs were recently published. The first described a woman with symptomatic hereditary transthyretin‐related amyloidosis who received patisiran, a siRNA silencing transthyretin (*TTR*) gene, during the third week of amenorrhea. Treatment was discontinued when pregnancy was confirmed, and the mother delivered a healthy baby after 40 weeks of gestation without any complications, developmental retardation or malformations [36]. The second report was on a woman with heterozygous familial hypercholesterolemia previously treated with inclisiran, a siRNA targeting proprotein convertase subtilisin/kexin type 9 (PCSK9). She received two doses of inclisiran, 25 and 13 weeks before pregnancy. The treatment was ceased when she became aware of the pregnancy and she delivered a healthy baby after 41 weeks of gestation [37].

Physiological modifications occurring during pregnancy may affect the absorption and distribution of siRNAs [38], and more data are required to assess their safety for both the mother and the fetus. siRNAs can be administered intravenously or subcutaneously (such as plozasiran), and this might have a different impact on pregnancies [38]. There is a 30%–50% increase in cardiac output during pregnancy which results in increased drug absorption [39, 40, 41]. It is also mandatory to evaluate the saturation of asialoglycoprotein receptor (ASGPR), the main receptor binding the GalNAc‐siRNAs, to avoid drug accumulation in the bloodstream and possible off‐target issues [38]. However, previous studies conducted in mouse models suggest that ASGPR activity could increase by two‐ to three fold during pregnancy [42, 43], which could possibly decrease siRNAs Cmax time in the bloodstream.

In the last decade, several successful pregnancies involving women with persistent chylomicronemia having previously been treated with lipid‐lowering biodrugs with long half‐lives have been published. One of these involved a woman treated with alipogene tiparvovec (Glybera), an AAV‐based intramuscular *LPL* gene replacement therapy. This patient was treated with Glybera 1 year before her first pregnancy which went well, without any abdominal pain or AP episode, and led to a normal delivery of a healthy baby [44].

Two pregnant women with sHTG who were using volanesorsen, an antisense oligonucleotide (ASO) targeting ApoC3, were reported. The first was a woman who ignored that she was pregnant until the 38th week of gestation and remained treated with monthly injections of volanesorsen during gestation. The pregnancy went well, and she delivered a healthy baby. The second woman was using volanesorsen for 5 years and she stopped this medication 6 months before conception. She had an AP episode at week 22 of gestation and volanesorsen was then reintroduced at week 23. She delivered a healthy baby at week 35 without any other AP episode [45].

Most monographs advise against pregnancy in patients requiring lipid‐lowering therapy, especially in patients with genetic dyslipidemia. It is very likely that no study will be conducted to this effect. The clinician must therefore rely on reports in addition to the pharmacokinetics of the molecule used. This case report adds to data having previously been collected from pregnant women treated with siRNA agents for different health conditions or from pregnancies in women with persistent chylomicronemia treated with oligonucleotide‐based treatments. The present report suggests that plozasiran might be safe for the mother and the fetus and could prevent incremental pregnancy‐driven TG elevation and occurrence of AP in women with severe HTG, even if it is administered 2 months before conception. This is consistent with the long duration of action and hepatic half‐life of plozasiran in clinical studies where TG levels remained sustainably lower than baseline > 9 months after the last injection [33, 34]. More data are needed to replicate this observation.

## Author Contributions

D.G. and M.L. contributed to the study design, data interpretation, and manuscript writing. All authors participated in data interpretation and critical review of the manuscript, had full access to all the data in the case report and approved this manuscript for publication.

## Funding

The clinical trial PALISADE (NCT05089084) was funded by Arrowhead Pharmaceuticals. However, the authors confirm independence from the sponsors; the content of the article has not been influenced by the sponsors.

## Ethics Statement

The clinical trial PALISADE (NCT05089084) has been approved locally on 30 November 2021 by Advarra (Pro00058009).

## Consent

The patient gave her informed consent to the submission and publication of this manuscript.

## Conflicts of Interest

M.L. received a PhD grant from Centre Intersectoriel en Santé Durable (CISD), Université du Québec à Chicoutimi. M.M. is employed by Arrowhead Pharmaceuticals. D.G. reports research grants or consulting fees from Amgen, Amryt, Arrowhead, CRISPRx, Eli Lilly, Ionis, Esperion, Merck, New Amsterdam pharma, Novartis, Regeneron, Ultragenyx, and Verve Therapeutics. D.G. research grants payments are received by ECOGENE‐21, an academic nonprofit research organization. N.R., D.B., C.G., and P.P. have nothing to disclose.

## Data Availability

The data that support the findings of this study are available on request from the corresponding author. The data are not publicly available due to privacy or ethical restrictions.
